# Genomic and functional characterization of a novel halophilic bacteriophage targeting carbapenem-resistant *Klebsiella pneumoniae*

**DOI:** 10.1371/journal.pone.0348054

**Published:** 2026-05-28

**Authors:** Sahar Abed, Masoumeh Beig, Sepideh Soltani, Mohadeseh Pahlevani, Peter Speck, Morvarid Shafiei, Abdolrazagh Hashemi Shahraki, Abozar Ghorbani

**Affiliations:** 1 Department of Microbial Biotechnology, Faculty of Basic Sciences and Advanced Technologies in Biology, University of Science and Culture, Tehran, Iran; 2 Department of Bacteriology, Pasteur Institute of Iran, Tehran, Iran; 3 College of Science and Engineering, Flinders University, Bedford Park, South Australia; 4 Division of Laboratory Services, Tennessee Department of Health, Nashville, Tennessee, United States of America; 5 Nuclear Agriculture Research School, Nuclear Science and Technology Research Institute (NSTRI), Karaj, Iran; Shahjalal University of Science and Technology, BANGLADESH

## Abstract

Carbapenem-resistant *Klebsiella pneumoniae* (CRKP) is a multidrug-resistant (MDR) pathogen causing severe infections in immunocompromised patients, prompting the exploration of alternative therapies like bacteriophage therapy. In this study, we isolated and characterized a novel halophilic lytic bacteriophage, Halo KS-7, targeting *K. pneumoniae*, and used an AI-driven annotation pipeline in Python to analyze its genome and therapeutic potential. Bacteriophages were isolated from Hospital wastewater, purified through plaque isolation, and confirmed using the double-layer agar method. Morphological analysis via transmission electron microscopy (TEM) and plaque assays assessed lytic activity. *In vitro* assays, including one‑step growth curve and MOI determination, were performed to evaluate the replication kinetics and lytic activity of bacteriophage Halo KS‑7 against carbapenem‑resistant Klebsiella pneumoniae. *In vivo* efficacy was assessed using a BALB/c mouse wound infection model by monitoring wound contraction and performing blinded histopathological analysis following phage treatment. DNA sequencing was done using Illumina HiSeq 2000, followed by genome assembly, AI-guided annotation, gene prediction, protein function classification, and comparative genomics using CLC Genomics Workbench. We also evaluated host range, temperature stability, pH sensitivity, and salt stress tolerance to assess therapeutic potential. Halo KS-7 exhibited strong lytic activity against CRKP and was classified as a Myoviridae bacteriophage by TEM. Phenotypic assays demonstrated optimal activity at 37 °C and neutral pH, effective activity from pH 4–10, and enhanced performance in high-salinity conditions. Bacteriophage Halo KS-7 exhibited a short latent period (~20 min), a modest burst size (5.73 PFU/cell), and optimal antibacterial activity at MOI 0.1, resulting in sustained suppression of *K. pneumoniae* growth *in vitro*. *In vivo*, Halo KS-7 treatment significantly enhanced wound healing in infected BALB/c mice, achieving near-complete wound closure, effective infection control, and improved histopathological regeneration comparable to uninfected controls. Halo KS-7 have 58.716 kb linear dsDNA genome (44.4% G + C), contains 49 predicted ORFs, lacks integrase, lysogeny, or antibiotic-resistance genes, and includes three tRNA genes (tRNATyr, tRNAPro, and tRNAAsn). It also includes a toxin gene and auxiliary factors like MazG, pyrophosphatase, and HNH endonucleases that enhance bacterial killing without promoting horizontal gene transfer or resistance. Functional annotation assigned ~65% of ORFs to structural, replication, and packaging roles. Comparative genomics showed moderate similarity to other Myoviridae but with distinct accessory features, emphasizing its novelty and therapeutic value. Halo KS-7 is a novel, strictly lytic bacteriophage with strong antibacterial activity and stress resilience, supporting its use as a promising biocontrol agent against CRKP and its potential for clinical development in managing MDR infections.

## 1. Introduction

Multidrug-resistant (MDR) *Klebsiella pneumoniae* is a major global health threat, causing various infections such as pneumonia and bloodstream infections [1,2]. Carbapenem-resistant *K. pneumoniae* (CRKP), a particularly concerning pathogen, has been classified as a critical priority by the World Health Organization due to its high antibiotic resistance and limited treatment options [3,4]. This bacterium’s ability to form biofilms, its high antibiotic resistance, and its potential for horizontal gene transfer (HGT) complicate treatment [5]. As traditional antibiotics become less effective, alternative therapeutic approaches are urgently needed [6].

Bacteriophages are emerging as a promising solution for antibiotic-resistant bacteria. They offer advantages like high specificity, targeting antibiotic-resistant strains, and minimal disruption to the host microbiota [7–9]. Some bacteriophages, particularly those targeting *K. pneumoniae*, also produce depolymerase that degrades the bacterial capsule, improving immune clearance and bacteriophage effectiveness [10,11].

Bacteriophages isolated from sources such as sewage and clinical samples may exhibit considerable genomic diversity. Continued isolation and genomic analysis of new bacteriophages are critical for advancing bacteriophage therapy and understanding bacteriophage-host–host interactions. In particular, bacteriophages from extreme environments such as high-salt (halophilic) conditions may offer unique features, including improved environmental or physiological stability and expanded host range [12].

Halophilic bacteriophages adapted to high-salt environments are highly stable under thermal, pH, and desiccation stress. Their unique genomes offer opportunities for genetic engineering and help improve our understanding of bacteriophage-host interactions. Notably, these bacteriophages also have potential practical uses, such as biocontrol agents in salt-rich food production and stable topical treatments for MDR infections under challenging environments like chronic wounds [13].

However, they remain underexplored in non-halophilic hosts like *K. pneumoniae*. Advances in artificial intelligence (AI) and bioinformatics have revolutionized bacteriophage research, enabling efficient genome annotation and identification of potentially useful therapeutic genetic elements in bacteriophages [14], such as holins, important for host lysis and MazG-like pyrophosphatases, which can neutralize host defenses to ensure virus [15].

While some studies have explored bacteriophages targeting MDR *K. pneumoniae*, few have systematically utilized computational pipelines to evaluate their genomic features and therapeutic relevance [16–18]. Limited bacteriophage databases and a lack of understanding of host specificity and infection mechanisms continue to hinder the advancement of bacteriophage therapy [18–20]. These gaps show the need for continued discovery and genomic investigation of novel bacteriophages, particularly those with unique ecological adaptations.

While CLC Genomics Workbench (Qiagen, Hilden, Germany) is a popular commercial tool known for its intuitive interface and integrated bioinformatics functionalities, it is limited in flexibility, transparency, and scalability, making it less suitable for researchers who need greater control over their workflows [21]. Python-based pipelines offer a powerful open-source alternative that allows for customization and transparency, enabling researchers to fine-tune their analysis, from preprocessing and quality filtering to genome assembly and annotation [22]. Python’s seamless integration with libraries like Biopython, pandas, matplotlib, and NumPy supports advanced data analysis, machine learning, and high-quality visualizations, offering greater precision and adaptability than commercial software [23]. Additionally, Python promotes reproducibility through script-based workflows and can be easily automated to handle large datasets without costly licenses [24]. Python’s scalability allows workflows to be deployed on various platforms, including local machines, high-performance clusters, and cloud infrastructure. Moreover, unlike proprietary tools with significant financial barriers, Python is cost-effective and freely available. Here, we used a Python-driven pipeline to characterize the novel halophilic bacteriophage Halo KS-7, demonstrating Python’s robustness, adaptability, and versatility of open-source solutions. This allows us to identify genes associated with therapeutic relevance. By comparing this genome to other known *K. pneumoniae* bacteriophages, we aim to identify distinctive traits that could support its potential as a biocontrol agent. This study seeks to demonstrate the value of integrating wet-lab techniques with computational tools in discovering and evaluating promising bacteriophage candidates. Both workflows were applied to the same dataset to ensure cross-validation of genome assembly and annotation results using independent algorithms, rather than to perform a performance comparison.

## 2. Materials and methods

### 2.1. Bacteriophage Isolation and Host Bacteria

Halo KS-7 was isolated from wastewater at Shahriar Hospital in Tehran, Iran. The sewage sample was centrifuged (10,000 × g, 10 min) and filtered through a 0.22 μm membrane. The host bacterium, *K. pneumoniae*, was cultured in Luria-Bertani (LB) broth at 37˚C until the logarithmic growth phase was reached. The wastewater sample (50 mL) was enriched with an equal volume of exponential-phase *K. pneumoniae* culture and incubated at 37°C for 24 hours with gentle agitation. The host *K. pneumoniae* isolates were carbapenem-resistant clinical strains previously isolated from diabetic foot ulcers of hospital patients.

The resulting bacteriophage lysate underwent purification via three successive cycles of single-plaque selection and co-culturing. Bacteriophage isolation was verified using the double-layer agar (DLA) method [25]. The plate was incubated at 37˚C for 24 h, and clear plaques indicated a successful bacteriophage infection. Bacteriophage titration involved tenfold serial dilutions in SM buffer (100 mM NaCl, 8 mM MgSO4, 50 mM Tris (pH = 7.5), and 0.002% gelatin (w/v)). Bacteriophage titers were determined by the double agar overlay plaque assay and reported as plaque-forming units per milliliter (PFU/mL) [5].

### 2.2. Morphological characterization by transmission electron microscopy (TEM)

Following standard protocols, isolated bacteriophage particles were analyzed using transmission electron microscopy (TEM) [26]. Briefly, 10 μL of purified bacteriophage suspension was applied to a carbon-coated copper grid and allowed to adsorb for 3–5 minutes. The sample was then negatively stained with 1% (w/v) uranyl acetate (pH = 7). Imaging was performed using a Zeiss LEO 906 TEM (Carl Zeiss LEO EM 906 E, Germany) operating at an accelerating voltage of 100 kV [27,28].

### 2.3. Host range determination

The host range of Halo KS-7 was evaluated on a panel of bacterial strains to assess its lytic spectrum. This panel included 30 CRKP strains [29]. Host range spot tests were conducted by spotting 10 μL of high-titer bacteriophage (~10^9 PFU/mL) onto lawns of each bacterial strain on appropriate agar. Plates were incubated overnight at 37 °C, and lysis was assessed qualitatively based on plaque morphology. Transparent or opaque plaques indicated susceptibility to Halo KS-7, reflecting lytic activity. In contrast, the absence of plaques or only faint clearing suggested resistance to the bacteriophage.

#### 2.3.1. Antimicrobial susceptibility test.

The susceptibilities of 30 *K. pneumoniae* isolates against 18 antibiotics including imipenem (IMP), ceftazidime (CAZ), cefotaxime (CTX), cefepime (CPM), piperacillin/tazobactam (PTZ), ampicillin/sulbactam (SAM), amoxicillin-clavulanate acid (AMC), imipenem (IMP), doripenem (DOR), meropenem (MEN), aztreonam (AZT), amikacin (AN), gentamicin (GM), ciprofloxacin (CIP), tetracycline (T), minocycline (MN), trimethoprim/Sulfamethoxazole (SXT), fosfomycin (FOS) obtained from the Mast Group Ltd., Merseyside, UK were tested by agar disk-diffusion method according to clinical and laboratory standards institute (CLSI, 2024) guidelines. The *E. coli* ATCC 25922 was used as a control for the disk diffusion method.

### 2.4. Physical and stability tests

The stability of Halo KS-7 was evaluated under a range of conditions representing physiological and extreme environments. To assess thermal stability, bacteriophage lysates (~10⁸ PFU/mL in SM buffer) were incubated for 1 hour at (−20°C, 4°C, 37°C, 50°C, 60°C, and 70°C), followed by titration using plaque assays on LB broth (Wang et al., 2016). For pH stability, aliquots were adjusted to pH values (2, 4, 7, 10, and 14) using SM buffer modified with HCl or NaOH and incubated at room temperature for 1 hour before titration. Halotolerance was assessed by incubating bacteriophage lysates in SM buffer supplemented with varying NaCl concentrations (5%, 10%, and 15%) at 25°C for 24 hours, followed by titer determination using the double agar layer (DLA) method [30]. All experiments were performed in triplicate.

### 2.5. One-step growth curve assay

The replication dynamics of bacteriophage Halo KS-7 were characterized using a one-step growth curve assay. A mid-exponential-phase culture of carbapenem-resistant CRKP was harvested by centrifugation and resuspended in fresh growth medium. The bacterial suspension was inoculated with Halo KS-7 phage lysate and incubated at 37 °C to allow phage adsorption. After adsorption, unbound phages were removed, and the infected cells were incubated under identical conditions. Samples were collected at 10-minute intervals for 120 min, and phage titers at each time point were quantified using the DLA method. The latent and rise periods were determined from the resulting growth curve, and the burst size was calculated as the mean number of progeny phages released per infected bacterial cell [27].

### 2.6. Multiplicity of infection determination

The multiplicity of infection (MOI) was determined to establish the optimal ratio of bacteriophage particles to bacterial host cells for efficient infection. This parameter is critical for assessing phage infectivity and replication efficiency. MOI determination was conducted by varying phage concentrations while maintaining a constant bacterial cell density, enabling controlled evaluation of phage–host interactions [27].

### 2.7. *In vivo* wound infection model and phage treatment

All animal experiments were performed in compliance with the ethical guidelines approved by the Ethics Committee of the Pasteur Institute of Iran, Department of Bacteriology (approval code: IR.PII.REC.1402.051). Five-week-old BALB/c mice (body weight 25 ± 2 g) were obtained from the Pasteur Institute of Iran and acclimated under laboratory conditions for one week before experimentation.

Mice were randomly allocated into four experimental groups (n = 5 per group): (i) healthy control, (ii) wounded negative control treated with sterile distilled water, (iii) positive control (wounded and infected, without treatment), and (iv) phage-treated group (wounded, infected, and treated with bacteriophage Halo KS-7). Anesthesia was induced via intraperitoneal injection of a ketamine/xylazine cocktail. Following shaving and aseptic preparation of the dorsal skin (vertebrae L2–L6), a standardized full-thickness excisional wound (5 mm in diameter) was created using a sterile biopsy punch, extending through the epidermis and superficial dermis while avoiding injury to the underlying musculature and minimizing bleeding.

Except for the negative control group, wounds were inoculated with 50 µL of a *K.pneumoniae* suspension adjusted to 1.5 × 10⁸ CFU/mL. After 24 h, infected wounds in the treatment group received bacteriophage Halo KS-7 at the optimal MOI = 0.1, as determined by *in vitro* assays, whereas infected wounds in the positive control group received no therapeutic intervention. Animals were housed individually under standard conditions, with ad libitum access to food and water and a 12 h light/dark cycle. Wound healing and infection progression were monitored for 14 days post-infection [27,28].

#### 2.7.1. Wound healing assessment.

The wound healing process was evaluated by photographing the wounds of each mouse and measuring wound dimensions on days 1, 3, 7, 10, and 14 following wound induction. Wound diameters were determined using digital calipers to ensure measurement accuracy and consistency across all time points. Healing progression was quantified by calculating the percentage of wound contraction using the equation below, where A_0_ denotes the initial wound area immediately after wound creation and A_T_ represents the wound area at the corresponding post-treatment time points [31]. Healing progression was quantified as the percentage of wound contraction, calculated using the following equation:


$$\begin{array}{c} \text{Wound contraction }(\%)=\left({\text{A}}_{\text{0}}-{\text{A}}_{\text{T}}\right)\times \text{100} \end{array}$$

### 2.8. Histopathological analysis

For histopathological evaluation, wound tissue samples were harvested from euthanized mice and processed according to standard histological protocols. Briefly, excised tissues were fixed in formalin, embedded in paraffin, sectioned, and stained with hematoxylin and eosin (H&E) for light microscopic examination. Histopathological assessments were conducted in a blinded manner by an experienced pathologist. Key parameters associated with wound healing and tissue remodeling—including hair follicle formation, angiogenesis, macrophage infiltration, fibroblast activity, collagen deposition, epidermal thickness, inflammatory response, and tissue regeneration—were systematically evaluated. A semi-quantitative scoring system, previously described in the literature [32], was employed to facilitate objective comparison of histopathological features among the different experimental groups [31].

### 2.9. Bacteriophage DNA extraction and genome sequencing

According to the manufacturer’s instructions, the bacteriophage genome was extracted using a commercial DNA extraction kit (DNA Pure, FAVOR-GEN, Iran). Quality and purity of the extracted DNA were verified through optical density measurements, agarose gel electrophoresis, and a PCR assay using bacterial 16S rRNA primers to confirm the absence of bacterial genomic contamination [33].

An Illumina library was constructed from 100 ng of purified Halo KS-7 DNA for whole-genome sequencing (WGS) using a Nextera XT Library Preparation Kit. Paired-end sequencing (2 × 150 bp) was carried out on the Illumina HiSeq platform. Raw sequencing reads were subjected to quality assessment using FastQC software, followed by adapter removal and quality filtering with Trimmomatic. High-quality reads were de novo assembled using SPAdes v3.15.2 with the “careful” mode enabled to reduce mismatches and short indels. Assembly quality was evaluated, resulting in a single contiguous bacteriophage genome. Any remaining gaps or ambiguous regions were resolved via Sanger sequencing of PCR-amplified fragments. The finalized genome was confirmed to be a linear double-stranded DNA molecule with defined termini, as determined by read coverage analysis and identification of putative terminal repeats [34].

### 2.10. Bioinformatics analysis

This study used two complementary approaches to analyze bacteriophage sequencing data. The first approach utilized an AI-enabled pipeline for viral detection and validation from next-generation sequencing data [35]. This pipeline integrates advanced bioinformatics tools with AI to efficiently identify viral sequences and de novo genome assembly, offering a comprehensive view of bacteriophage genetic diversity. The second approach involved using CLC Genomics Workbench 22, a widely adopted platform that supports read processing, mapping, variant calling, and genome assembly through an intuitive graphical interface and robust analytical capabilities.

#### 2.10.1. AI-driven genome annotation pipeline.

In this study, the WGS data of bacteriophage Halo KS-7 were analyzed using a previously published and validated bioinformatics pipeline described by Ghorbani et al. (2024) [35]. This pipeline provides an automated and reproducible workflow for sequencing read processing, de novo genome assembly, genome annotation, and functional characterization, as detailed in the original publication. The de novo genome assembly of Halo KS-7 was generated from a total of 9,028,826 sequencing reads, resulting in an N50 of approximately 5,000 bp. Coverage analysis indicated 3,969,279 reads in high-coverage regions (30 signatures) and 3,969,279 reads in low-coverage regions (22 signatures), yielding a total of 52 coverage signatures across the genome. These metrics confirm sufficient sequencing depth and uniform coverage for reliable genome assembly and annotation. Genome annotation and comparative genomic analyses were conducted within the pipeline using established bioinformatic tools and reference databases to identify open reading frames, conserved domains, and taxonomic relationships.

All computational analyses were executed using the Python scripts provided by the referenced study, ensuring consistency with established methodologies. All analyses were performed using the default parameter settings of the pipeline, and no modifications were made to the underlying code or tool configurations.

#### 2.10.2. Analysis using CLC genomics workbench 22.

CLC Genomics Workbench was used as an independent validation platform to confirm genome structure, ORF predictions, and annotation consistency obtained from the Python-based AI pipeline. Sequencing reads were trimmed using CLC Genomics Workbench 22 (QIAGEN) with standard parameters to remove adapter sequences, ambiguous nucleotides, and low-quality bases. Specifically, bases with quality scores ≤5, reads shorter than 15 nucleotides and reads containing more than two ambiguous nucleotides were removed [36]. The cleaned reads were assembled de novo using default parameters (word size: 15 nt).

Assembled contigs were subjected to nucleotide BLAST analysis using Geneious version 22 (Biomatters, New Zealand). The contig with the highest similarity to viral sequences in the GenBank database was selected for further study to identify putative bacteriophages. Confirmation was performed using the VirusDetect tool (http://virusdetect.feilab.net/cgi-bin/virusdetect/vdo_home.cgi), and results were independently validated by mapping cleaned reads back to the viral genome identified in the BLAST results.

Genome annotation was carried out using the CLC Microbial Genomics Module, leveraging a reference genome and integrated BLAST tools. Annotation parameters included a default similarity threshold of 95% and an E-value cutoff of 0.0001 [36]. The finalized genome sequence was then aligned with selected bacteriophage genomes from the NCBI database using the Whole Genome Alignment plugin in CLC Genomics Workbench [37], providing insights into phylogenetic relationships and genomic conservation.

### 2.11. Inclusivity in global research

Additional information regarding the ethical, cultural, and scientific considerations specific to inclusivity in global research.

## 3. Results

### 3.1. Isolation and characterization of a halophilic *K. pneumoniae* bacteriophage

**Fig 1:**
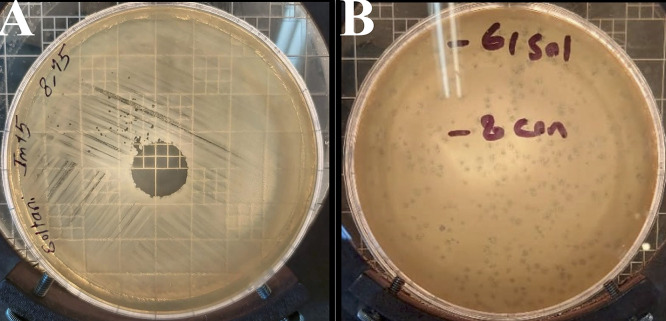
Lytic activity of bacteriophage Halo KS-7 against *Klebsiella pneumoniae.* **(A)** Spot test showing a clear lysis zone on a bacterial lawn. **(B)** Plaque assay revealing discrete plaques formed by Halo KS-7.

**Fig 2:**
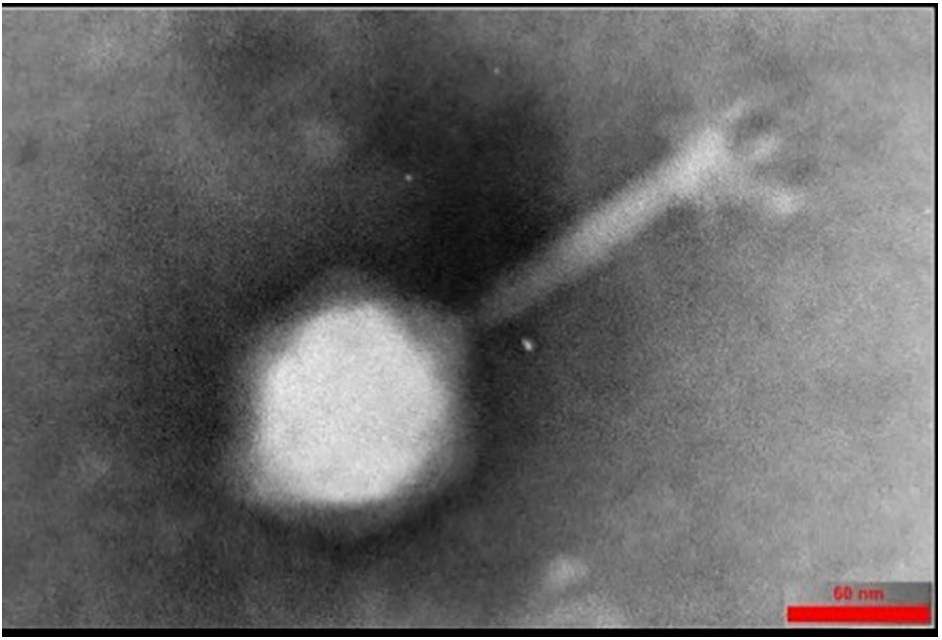
Electron microscopy analysis of Halo KS-7 bacteriophage morphology. Transmission electron micrograph of the Halo KS-7 bacteriophage, negatively stained with 2% (w/v) uranyl acetate. The image highlights the bacteriophage’s characteristic structure, including the head and tail, with a scale bar representing 60 nm.

The Halo KS-7 bacteriophage was specifically isolated to target CRKP. The spot assay results, illustrated in Fig 1A, and the DLA assay outcomes presented in Fig 1B unequivocally affirmed its lytic activity. TEM analysis revealed that Halo KS-7 exhibits a morphology consistent with the *Caudovirales* order (Fig 2).

### 3.2. Host range determination

Halo KS-7 exhibited a broad host range across the tested CRKP isolates. In assays involving 30 clinical CRKP strains, clear plaques were observed in 57% of the strains, confirming the bacteriophage’s selective and targeted lytic activity.

#### 3.2.1. The results of the disk diffusion test.

Disk diffusion method revealed that the resistance rates of the antimicrobial agents were as follows: IMP 100% (30/30), CTX 77% (23/30), CAZ 67% (20/30), SAM 27% (8/30), IMP 57% (17/30), FOS 57% (17/30), CPM 57% (17/30), T 57% (17/30), AZT 50% (15/30), MEN 53% (16/30), AMC 43% (13/30), SXT 33% (10/30), AN 37% (11/30), CIP 25% (10/40), DOR 22% (9/40), PTZ 22% (9/40), GM 22% (9/40), and MN 12% (5/40).

### 3.3. Physical and stability tests

#### 3.3.1. Temperature stability.

**Fig 3:**
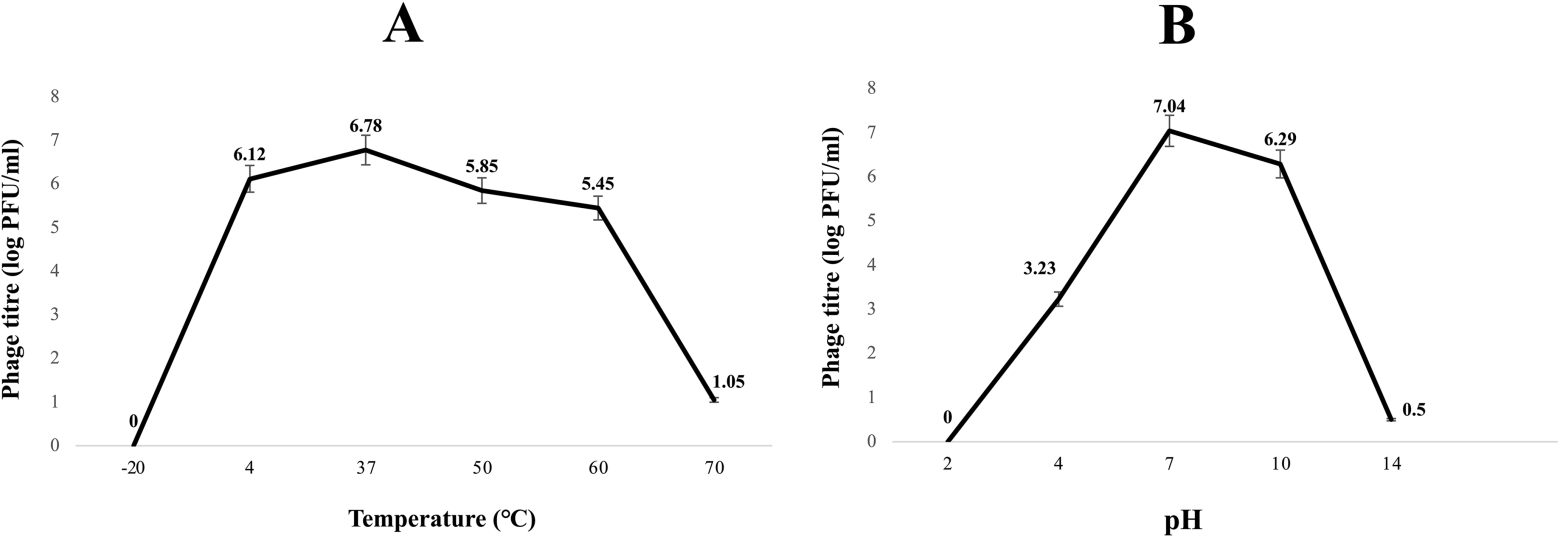
Stability of Halo KS-7 bacteriophage under different temperature and pH conditions. **(A)** Thermal stability of the bacteriophage assessed at various temperatures ranging from −20 °C to 70 °C. The highest bacteriophage titre was observed at 37 °C, significantly decreasing at extreme temperatures. **(B)** pH stability profile of the bacteriophage evaluated across a pH range of 2 to 14. Optimal bacteriophage stability was recorded at pH = 7, with reduced titres under highly acidic and alkaline conditions. Bacteriophage titres are expressed as log PFU/ml. Error bars represent the standard deviation from triplicate experiments.

Fig 3A illustrates the thermal stability of Halo KS-7. The bacteriophage exhibited optimal lytic activity at 37°C and maintained good activity in the range of 4°C to 60°C, with no significant drop in titer (within 0.5 log of the initial). At 70°C, a notable decrease in lytic activity was observed. However, it did not reach complete inactivation, indicating that Halo KS-7 has moderate thermal stability, similar to many colibacteriophages but slightly lower than some extremophilic bacteriophages.

#### 3.3.2. pH sensitivity.

The influence of pH on Halo KS-7 bacteriophage’s lytic activity is seen in Fig 3B. The bacteriophage has good lytic activity at pH between 4 and 10; its highest activity was observed at pH = 7. At pH below 4, bacteriophage activity was completely stopped (pH = 2, no activity). As pH increased and the environment became more alkaline, the lytic activity of the bacteriophage decreased so that at pH = 14, its activity reached almost zero. At pH = 10, bacteriophage activity was still relatively high but decreased compared to its optimal value at pH = 7. These data indicate that the bacteriophage is most stable and effective in neutral and slightly alkaline conditions (pH = 7–10), while it is extremely vulnerable in highly acidic (pH = 2) and highly alkaline (pH = 14) environments.

#### 3.3.3. Salt tolerance.

**Fig 4:**
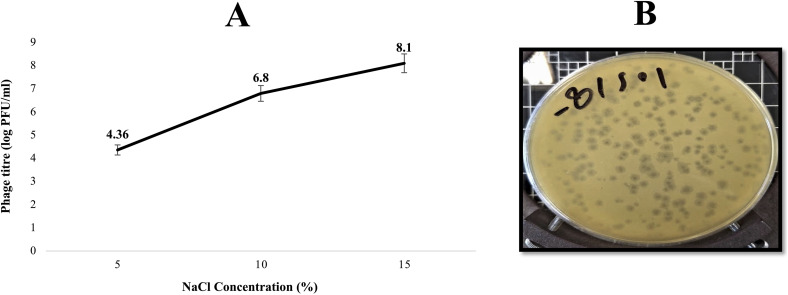
Effect of NaCl concentration on the stability and infectivity of Halo KS-7 bacteriophage. **(A)** Bacteriophage titre (log PFU/ml) measured after incubation at different NaCl concentrations (5%, 10%, and 15% w/v). The results indicate enhanced bacteriophage stability with increasing salinity, with the highest titre observed at 15% NaCl. Error bars represent standard deviations from triplicate experiments. **(B)** Plaque formation assay showing the lytic activity of Halo KS-7 on a host lawn, confirming infectivity under high-salt conditions.

Fig 4A depicts the response of Halo KS-7 to varying salt concentrations. The bacteriophage exhibited increased lytic activity as NaCl concentration in the medium rose. At 5% NaCl, the bacteriophage titer was 4.36 log PFU/mL, which increased to 6.8 log PFU/mL at 10% NaCl. The highest activity, 8.1 log PFU/mL, was at 15% NaCl.

These findings suggest that elevated NaCl concentrations led to the formation of larger, sharper plaques (Fig 4B), showing the beneficial effect of salt on bacteriophage lytic activity. Accordingly, Halo KS-7 is classified as a halophilic bacteriophage, demonstrating optimal lytic activity in saline environments.

### 3.4. One-step growth curve analysis

**Fig 5:**
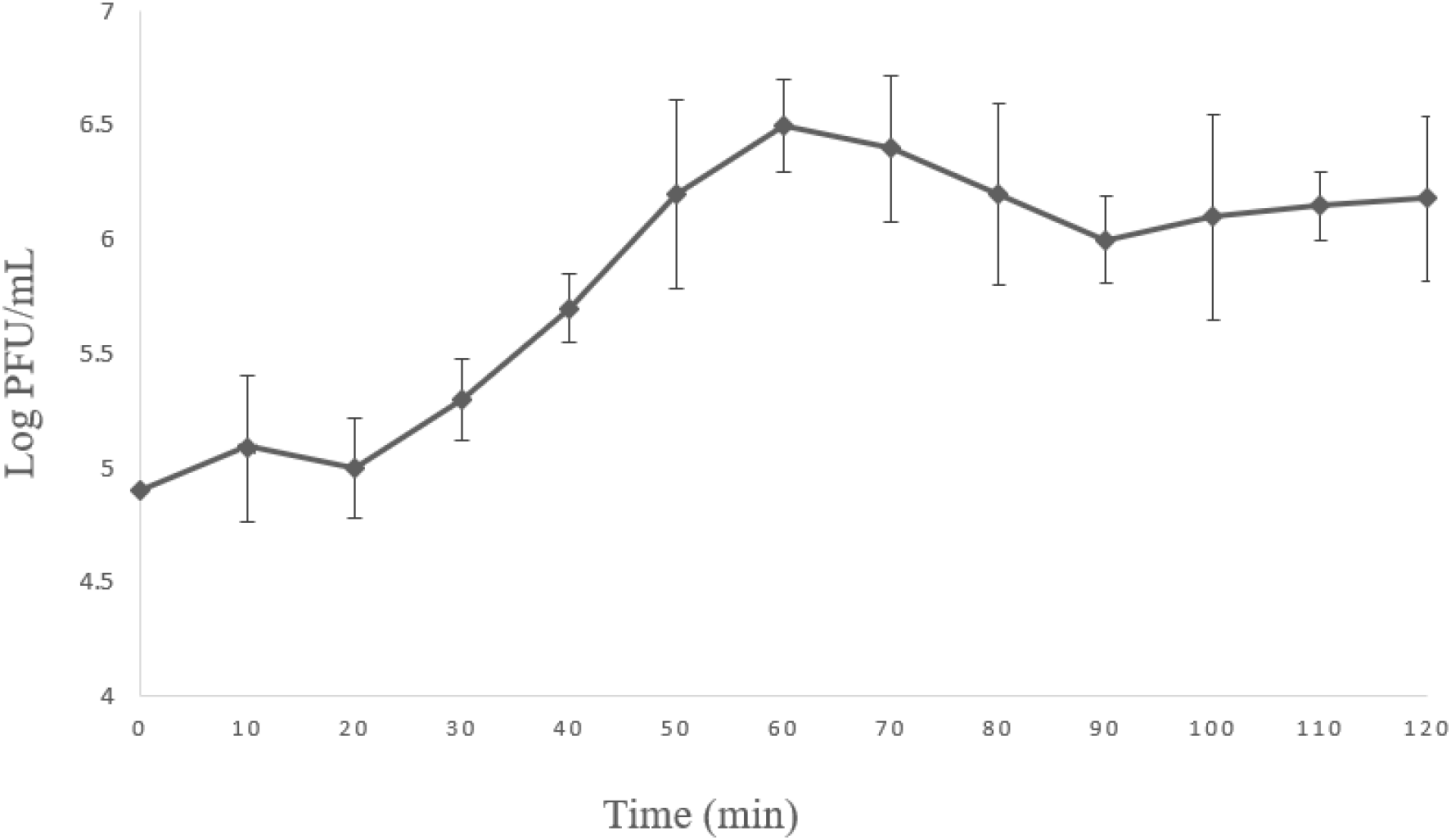
One-step growth curve of bacteriophage Halo KS-7 infecting Klebsiella pneumoniae. Phage titers (log PFU/mL) were determined at 10-minute intervals over 120 minutes using the double-layer agar method. The curve demonstrates a latent period of approximately 20 minutes, followed by a rise period and a subsequent plateau phase. The calculated average burst size was 5.73 PFU per infected bacterial cell. Data points represent mean values ± standard deviation from three independent experiments.

One-step growth curve analysis was performed to delineate the lytic replication kinetics of bacteriophage Halo KS-7 during infection of CRKP over a 120-minute post-infection period (Fig 5). The growth curve demonstrated a clearly defined latent period of approximately 20 min, followed by a rapid rise phase reflecting synchronized phage replication and host cell lysis. Phage titers subsequently reached a plateau, indicating completion of the lytic cycle and exhaustion of susceptible host cells. The mean burst size of Halo KS-7 was determined to be 5.73 plaque-forming units (PFU) per infected bacterial cell. Collectively, these data reveal a relatively short latent period and modest burst size, providing a quantitative framework for understanding the replication efficiency of Halo KS-7 and supporting genomic predictions of its strictly lytic life cycle.

### 3.5. Determination optimal multiplicity of infection

**Fig 6:**
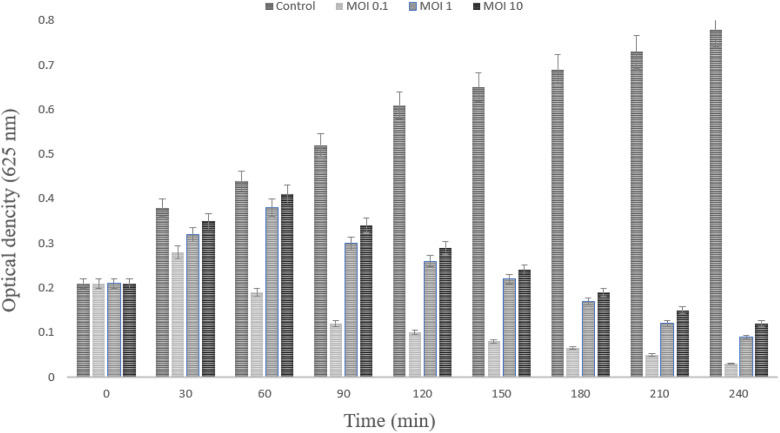
Multiplicities of infection (MOIs) determination experiment of Halo KS-7 phage against *K. pneumoniae.* Effect of different multiplicities of infection (MOIs) of bacteriophage Halo KS-7 on the growth of *Klebsiella pneumoniae*, measured as OD₆₂₅ over 240 min. All phage treatments inhibited bacterial growth compared with the control, with MOI 0.1 producing the most sustained reduction in optical density.

The influence of bacteriophage Halo KS-7 at different multiplicities of infection (MOIs) on the growth kinetics of *Klebsiella pneumoniae* was evaluated by measuring optical density at 625 nm over a 240-min incubation period (Fig 6). At the initial time point (0 min), all experimental groups, including the untreated control and phage-treated cultures, exhibited comparable optical density values, confirming equivalent starting bacterial concentrations.

In the absence of phage treatment, the control culture demonstrated a continuous and time-dependent increase in optical density throughout the experiment, rising steadily from approximately 0.21 at 0 min to nearly 0.78 at 240 min, consistent with logarithmic bacterial growth under the experimental conditions. In contrast, cultures exposed to Halo KS-7 at all tested MOIs (0.1, 1, and 10) showed a clear inhibition of bacterial growth beginning as early as 30–60 min post-infection. At an MOI of 0.1, a slight increase in optical density was observed during the early phase (30–60 min), suggesting initial bacterial replication before extensive phage amplification. This was followed by a pronounced and sustained decline in optical density from 90 min onward, reaching the lowest values among all treatment groups at later time points (180–240 min). This pattern indicates effective phage propagation, repeated infection cycles, and progressive lysis of the bacterial population over time. Phage treatment at higher MOIs (1 and 10) also resulted in significant suppression of bacterial growth relative to the control. However, although these conditions reduced optical density earlier in the experiment, bacterial OD values remained consistently higher than those observed at MOI 0.1 during the later stages. At MOI 10, in particular, a gradual decrease in optical density was followed by stabilization rather than continued decline, suggesting less sustained bacterial killing compared to the MOI 0.1 condition.

### 3.6. *In vivo* wound healing assessment

**Fig 7:**
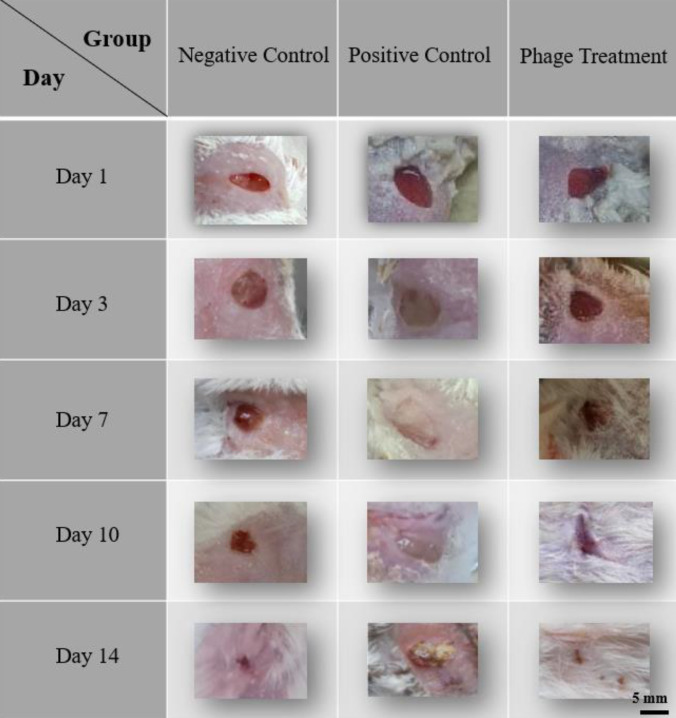
In vivo wound healing efficacy of bacteriophage Halo KS-7 in a *Klebsiella pneumoniae*–infected mouse model. Representative macroscopic images showing wound healing progression in BALB/c mice over 14 days following full-thickness excisional wound creation (~5 mm). Images were recorded on days 1, 3, 7, 10, and 14 post-wounding. Experimental groups included a negative control (wounded, uninfected), a positive control (wounded and infected with *K. pneumoniae* without treatment), and a phage-treated group (wounded, infected, and treated with Halo KS-7 bacteriophage). The negative control group exhibited normal wound contraction and near-complete closure by day 14. The positive control group showed persistent inflammation, exudate formation, delayed contraction, and incomplete wound closure throughout the study period, with no evident hair regrowth. In contrast, the Halo KS-7–treated group demonstrated accelerated wound contraction beginning at day 3, significant reduction in wound area by day 7, and substantial re-epithelialization by day 10. By day 14, phage-treated wounds were nearly completely healed, with visible hair regrowth and absence of overt infection. Scale bar: 5 mm.

Macroscopic evaluation of wound healing revealed clear differences among the experimental groups throughout the 14-day observation period (Fig 7). On day 1, all wounded animals exhibited comparable full-thickness excisional wounds (~5 mm in diameter), confirming consistent wound induction across groups.

In the negative control group (wounded, uninfected), wounds showed a normal and progressive healing pattern characterized by gradual contraction and scab formation, resulting in near-complete wound closure by day 14. In contrast, the positive control group (infected and untreated) exhibited pronounced signs of infection, including persistent inflammation, exudate accumulation, and delayed tissue repair. These wounds showed minimal contraction over time, incomplete closure, and impaired re-epithelialization, remaining visibly inflamed with necrotic or purulent tissue evident even at day 14. No apparent hair regrowth was observed in this group, indicating disrupted skin regeneration due to sustained bacterial infection.

Notably, wounds treated with bacteriophage Halo KS-7 displayed a markedly improved healing progression compared with the positive control group. Early signs of wound contraction were evident as early as day 3, followed by a pronounced reduction in wound size by day 7. By day 10, phage-treated wounds demonstrated substantial re-epithelialization and tissue regeneration, with diminished inflammation. By day 14, wounds in the phage-treated group showed near-complete closure, restoration of normal skin architecture, and visible hair regrowth, with no overt signs of local infection. Overall, the healing profile of the phage-treated group closely resembled that of the negative control group, indicating effective bacterial clearance and promotion of wound repair by Halo KS-7 *in vivo*.

#### 3.6.1. *In vivo* evaluation of wound healing following phage therapy.

**Fig 8:**
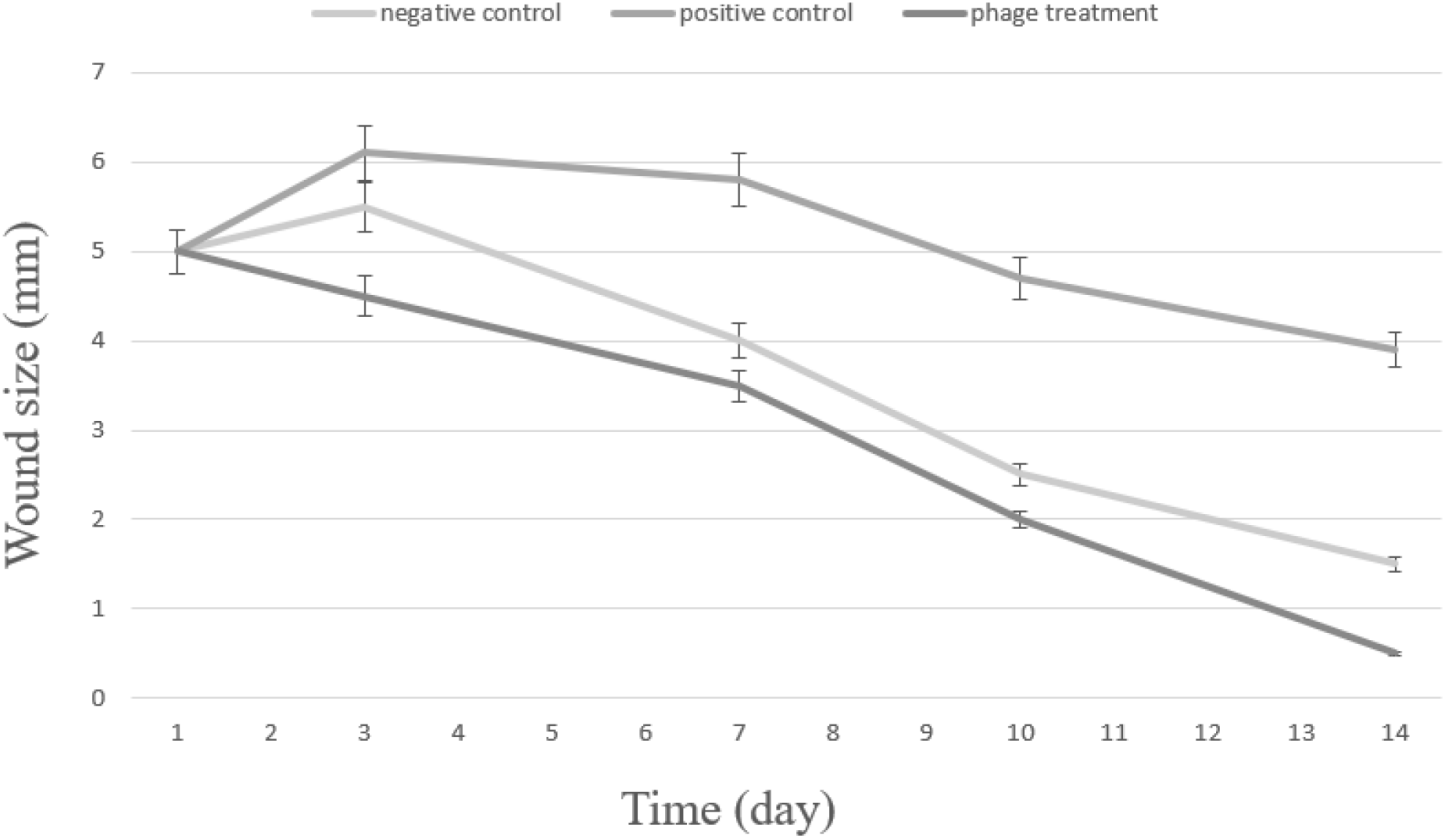
*In vivo* effect of Halo KS-7 bacteriophage therapy on wound healing in a *Klebsiella pneumoniae*–infected BALB/c mouse model. Full-thickness excisional wounds were monitored over 14 days in three experimental groups: negative control (wounded but uninfected), positive control (wounded and infected with *K. pneumoniae* without treatment), and phage-treated group (wounded, infected, and treated with Halo KS-7 bacteriophage). Wound size (mm) was measured at the indicated time points. All groups exhibited comparable initial wound sizes on day 1. The positive control group showed delayed wound closure and sustained tissue damage, whereas phage-treated wounds displayed rapid and progressive contraction, approaching complete closure by day 14. Data are presented as mean ± standard deviation (SD).

Fig 8 depicts the temporal progression of wound healing in BALB/c mice over 14 days following bacteriophage treatment, comparing three experimental groups: negative control (wounded but uninfected), positive control (wounded and infected with *K. pneumoniae* without treatment), and the phage-treated group. All groups exhibited comparable initial wound diameters of approximately 5 mm on day 1, confirming baseline uniformity before treatment.

In the negative control group, wounds underwent a typical physiological healing process. A slight transient increase in wound size was observed by day 3 (≈5.5 mm), likely reflecting the early inflammatory phase of wound repair. This was followed by sustained wound contraction, with dimensions decreasing to 4 mm on day 7, 2.5 mm on day 10, and reaching near-complete closure by day 14 (≈1.5 mm).

In contrast, the positive control group demonstrated markedly impaired wound healing consistent with ongoing bacterial infection. Wound size increased substantially during the early phase, peaking at approximately 6.1 mm on day 3, indicative of infection-associated inflammation and tissue damage. Although gradual contraction was observed thereafter, healing remained significantly delayed, with wounds measuring 5.8 mm, 4.7 mm, and 3.9 mm on days 7, 10, and 14, respectively. These findings indicate persistent infection and incomplete tissue repair in the absence of therapeutic intervention.

Notably, the phage-treated group exhibited a significantly accelerated and sustained wound healing response. Following phage administration, wound size decreased from 5 mm on day 1 to 4.5 mm by day 3, followed by a pronounced reduction to 3.5 mm on day 7 and 2.0 mm on day 10. By day 14, wounds were almost completely resolved, with residual wound sizes of approximately 0.5 mm. In addition to rapid wound contraction, phage-treated wounds showed clear signs of effective infection control, enhanced tissue regeneration, and visible hair regrowth—features that were absent in the positive control group.

Collectively, these results demonstrate that bacteriophage therapy effectively controls *K. pneumoniae* infection *in vivo* and significantly promotes wound healing. Importantly, phage treatment not only restored the healing trajectory disrupted by infection but resulted in wound closure kinetics comparable to, or exceeding, those observed in uninfected controls, highlighting the therapeutic potential of bacteriophage Halo KS-7 in infected wound management.

### 3.7. Histopathological analysis of wound healing

**Fig 9:**
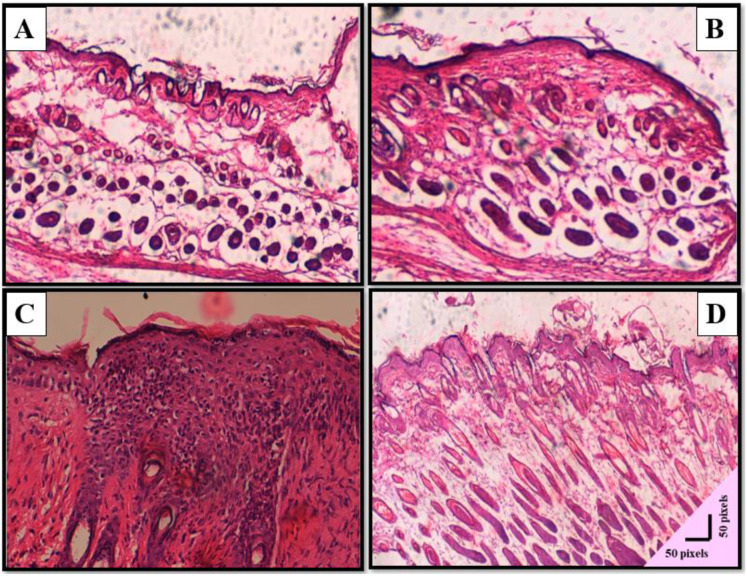
Histopathological assessment of wound healing. Representative hematoxylin and eosin **(H&E)**–stained skin sections (×10 magnification) from different experimental groups. **(A) Healthy control mice**, showing intact epidermal architecture with normal stratification and well-organized dermal layers. **(B) Negative control group** (wounded, uninfected, untreated), displaying partial re-epithelialization with moderate epidermal thickness and ongoing dermal remodeling. **(C) Positive control group** (wounded, *Klebsiella pneumoniae*–infected, untreated), characterized by severe tissue disruption, marked inflammatory cell infiltration, thickened hyperplastic epidermis, and delayed wound healing. **(D) Phage-treated group** (wounded, *K. pneumoniae*–infected mice treated with Halo KS-7 bacteriophage), demonstrating pronounced re-epithelialization, organized collagen deposition, reduced inflammation, and restoration of near-normal skin architecture, comparable to the uninfected controls. Scale bar: 50 µm.

Histopathological examination of wound tissues at day 14 revealed distinct healing responses across the experimental groups (Fig 9 and Table 1). The healthy control group exhibited normal skin architecture, with well-formed hair follicles, intact epidermal thickness, minimal inflammation, and advanced tissue regeneration, reflecting the natural healing process.

**Table 1:** Histopathological evaluation of wound healing in BALB/c mice on day 14 after *K. pneumoniae* infection and Halo KS-7 phage therapy.

Parameter	Healthy Control	Negative Control	Positive Control	Phage Treatment
Hair Follicle	+5	+4	+1	+3
Angiogenesis	0	+4	+1	+4
Macrophage	+1	+3	+5	+2
Density	+1	+3	+5	+2
Fibroblast Activity	+1	+3	+5	+2
Collagen	+1	+3	+4	+2
Epidermal Thickness	+5	+2	0	+1
Inflammation	+1	+3	+5	+2
Tissue Generation	+5	+4	+3	+5

Scores represent semi-quantitative histopathological grading, where 0 indicates absence or normal tissue appearance, and increasing scores (+1 to +5) denote progressively greater presence or intensity of the indicated parameter, reflecting the extent of inflammation, tissue remodeling, and regeneration.

In the negative control group, moderate repair was observed. This group showed increased angiogenesis, fibroblast activity, collagen deposition, and partial restoration of the epidermal structure, suggesting a moderate healing response despite the absence of infection.

Conversely, the positive control group displayed pronounced pathological changes indicative of delayed healing and persistent infection. These included intense inflammatory cell infiltration, elevated fibroblast activity, excessive collagen deposition, and a lack of epidermal thickening. Additionally, hair follicle regeneration and angiogenesis were significantly reduced, highlighting the adverse effects of untreated infection on wound healing.

Interestingly, the phage-treated group demonstrated a more balanced histological profile. There was a notable enhancement in angiogenesis and tissue regeneration, with reduced inflammation and controlled fibroblast activity and collagen deposition. Hair follicle regeneration was evident, and the epidermis began to restore, suggesting effective infection control and improved wound repair.

### 3.8. Whole genome sequencing and bioinformatics analysis: genome sequencing and assembly

**Fig 10:**
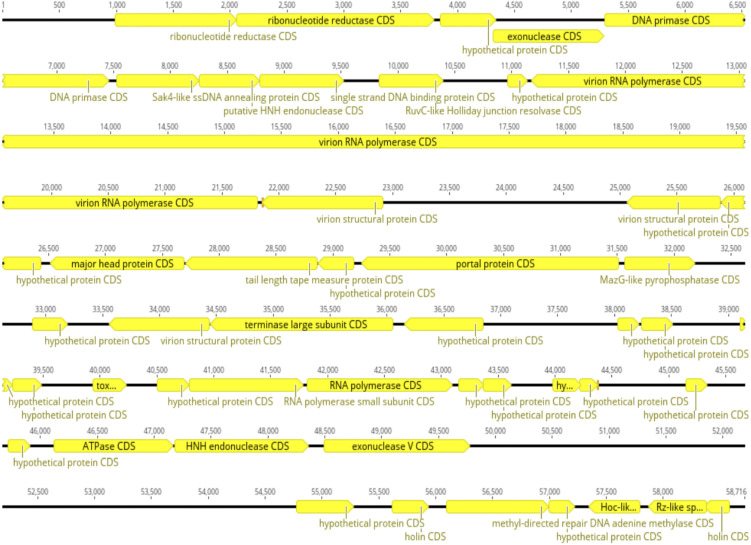
Whole-genome sequencing and bioinformatic analysis of Halo KS-7 bacteriophage. Annotated genome map of the Halo KS-7 bacteriophage based on the finalized genome annotation generated using the Python-based AI-driven pipeline and independently validated with CLC Genomics Workbench. The genome consists of 49 predicted ORFs, depicted as arrows indicating the direction of transcription. Functional annotation categorized ORFs into proposed modules, including replication, structural assembly, packaging, lysis, and hypothetical proteins. ORFs encoding proteins with conserved domains—such as DNA primase, RNA polymerase, terminase subunits, portal protein, and holins—are highlighted, while remaining ORFs were annotated as hypothetical due to the absence of significant homology. Only annotations concordant between both analytical approaches were retained in the final genome map.

A commercial column-based DNA extraction protocol ensured material purity without bacterial genomic contamination. The genome sequence data were assembled de novo. The complete genome sequence of Halo KS-7 has been deposited in GenBank under accession number PV026551. The Halo KS-7 bacteriophage genome has a genome size of 58,716 bp. It comprises 49 predicted ORFs, with 64.47% related to genes encoding putative functional proteins. The GC content of Halo KS-7 is 44.4%, and its closest related organism is *Klebsiella* bacteriophage vB_KpP_FBKp27 (accession number: NC_068555), with a 90.81% identity and 94% query coverage (Fig 10).

### 3.9. Functional annotation and genomic analysis

**Fig 11:**
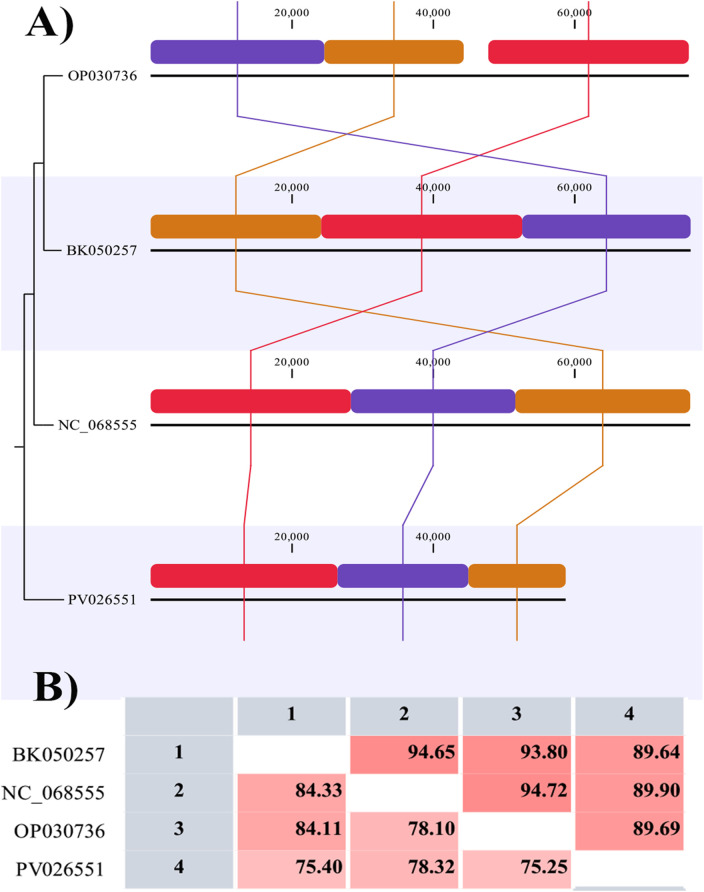
Comparative genomic and nucleotide identity analysis of Halo KS-7 and related bacteriophages. **(A)** Whole-genome alignment of Halo KS-7 and related bacteriophages generated using the Whole-Genome Alignment module in CLC Genomics Workbench. Colored blocks represent homologous alignment segments identified across the compared genomes. Colors are automatically assigned by the software to visualize corresponding regions of sequence similarity and genome collinearity and do not represent functional gene categories. **(B)** Heatmap showing pairwise average nucleotide identity (ANI) values among the compared genomes.

Fig 11A presents a comparative genomic alignment of multiple *K. pneumoniae* strains, showing the organization of genomic features across different isolates. The diagram shows colored bars representing various genomic regions, with each strain labeled on the left (e.g., OX335406, OP030736, BK050257, PV026551, and NC_068555). The connecting lines between the bars indicate similarity and homology between these genomic regions across strains, with each segment representing specific gene sequences or other functional genomic features. The scale on the top indicates the genomic coordinates, ranging from 0 to 60,000 base pairs. This comparison illustrates conserved and variable genomic regions across the strains, providing insights into their genomic diversity and evolutionary relationships. The visualization is crucial for identifying homologous gene clusters and potential regions of genetic variation and understanding the genomic structure of *K. pneumoniae* isolates.

Fig 11B provides a heatmap showing pairwise genome similarity percentages. The ANI values confirm that Halo KS-7 (PV026551) shares only moderate sequence identity (75.42%–78.34%) with its closest relatives. The comparison with NC_068555, which has the highest alignment score (90.81% identity but only 94% query coverage), suggests that Halo KS-7 is genetically distinct. Syntactic analysis and ANI calculations demonstrate that Halo KS-7 exhibits significant genetic divergence from known bacteriophages. The unique ORFs and moderate sequence similarity with its closest relatives strongly support its classification as a novel bacteriophage. Holin and spanin genes associated with host cell lysis were detected among the functional genes identified. Genes encoding HNH endonucleases and exonucleases were present, along with a RuvC-like Holliday junction resolvase and a Sak4-like single-stranded DNA annealing protein. A MazG-like pyrophosphatase, single-stranded DNA-binding proteins, ribonucleotide reductase, ATPase, and RNA polymerase genes were also identified. These functional genes collectively suggest a genetic repertoire potentially supporting bacteriophage replication, survival, and host interaction under various environmental conditions.

Halo KS-7’s genome encodes three tRNA genes (tRNA^Tyr^, tRNA^Pro^, and tRNA^Asn^). These likely enhance translational efficiency by supplementing the host’s tRNA pool, particularly when specific tRNAs are in short supply during infection.

### 3.10. Therapeutic potential and genetic safety considerations

Genomic analysis indicates that Halo KS-7 is exclusively lytic and lacks lysogenic properties, as well as genes associated with antibiotic resistance or virulence factors that make it an excellent candidate for therapeutic applications. Identifying key genes involved in cell wall degradation, adaptation to high-salinity environments, and enhanced bacteriophage efficacy underscores its potential to combat antibiotic-resistant infections. Some of the Halo KS-7 bacteriophage genes and their functional roles in therapeutic applications are shown in Table 2.

**Table 2:** Key functional genes in Halo KS-7 bacteriophage and their potential roles in therapy.

Gene Name	Function	Benefit of Bacteriophage Therapy	Reference
Holin CDS	Creates pores in the bacterial membrane, allowing endolysins to access the peptidoglycan.	Enhances bacterial lysis efficiency, accelerating infection.	[60]
Rz-like spanin CDS	Facilitates the final step of cell lysis by disrupting the outer membrane.	Ensures complete bacterial lysis, preventing persistence.	[61]
MazG-like pyrophosphatase CDS	Inhibits bacterial stress response by degrading nucleotide alarmones.	Reduces bacterial resistance mechanisms. Ensures the survival of bacteriophages within the bacterial cell.	[15]
RNA polymerase CDS	Transcribes bacteriophage genes independently of host machinery.	Enables efficient viral gene expression even in stressed hosts.	[62]
ribonucleotide reductase CDS	Converts ribonucleotides to deoxyribonucleotides for DNA synthesis.	Supports rapid bacteriophage replication in nutrient-limited conditions.	[59]

Genome analysis of Halo KS-7 revealed a putative toxin gene located between nucleotide positions 39,942 and 40,232, encoding a 96 amino acid protein (MAYQNNSAASRNNNTSSANAGEAPERNIGGYLNIGVRGRDGQVRRLGQGGRGIALREDHAVEGKVLEFLRAQGIEALDEHLVITFGDAKALENFEL). The amino acid sequence derived from the gene exhibited similarity to a toxin found in *Klebsiella*, as indicated by BLASTp analysis. However, the toxin could not be categorized into a specific family. Structural analysis using InterProScan identified a colicin-like motif within the protein sequence, which is typically associated with pore-forming activity and bactericidal effects.

## 4. Discussion

In this study, we identified Halo KS-7 as a novel halophilic bacteriophage infecting *K. pneumoniae*, with several characteristics distinguishing it from previously described bacteriophages. Notably, its strictly lytic nature and halotolerance provide advantages in therapeutic contexts, especially in environments where high salt concentrations may otherwise inhibit other bacteriophage activities. The bacteriophage’s genome encodes various auxiliary enzymes, including holins and spanins, which may enhance its ability to lyse bacterial cells efficiently.

Another notable feature is the presence of an auxiliary metabolic gene, MazG, encoded by gene gp_39. Auxiliary metabolic genes are typically of host origin and are co-opted by bacteriophages to manipulate host metabolic pathways in ways that support viral replication. MazG genes have been predominantly identified in marine bacteriophages and are associated with lytic and temperate lifestyles [38]. They are thought to facilitate bacteriophage propagation in nutrient-limited environments by modulating host nucleotide metabolism, thus aiding replication in starved cells [39]. Given this context, the presence of MazG in Halo KS-7 suggests it may possess the capacity to alter host metabolism and potentially interfere with host bacteriophage defense systems. However, further investigation is required to elucidate the specific mechanisms involved in Halo KS-7 ‘s interaction with its host.

A toxin-like gene in the lytic Halo KS-7 bacteriophage has significant implications for both its biological function and potential therapeutic applications. Unlike temperate bacteriophages, which can integrate their genomes into host bacteria and enter a lysogenic state, Halo KS-7’s strictly lytic nature ensures that it does not undergo lysogenic conversion or genome integration. This characteristic significantly reduces the risks typically associated with temperate bacteriophages, such as the potential HGT of toxin genes, which could raise biosafety concerns.

Recent studies challenge the long-held assumption that strictly lytic bacteriophages operate in predictable and straightforward ways, especially *in vivo*. Evidence suggests that even these bacteriophages may exhibit unexpected behaviors, such as host-switching or direct interaction with mammalian immune cells, which could have critical implications for bacteriophage therapy [40]. Moreover, some lytic bacteriophages appear capable of mediating HGT, including the mobilization of virulence factors, raising potential safety concerns. Bacteriophage capsids can also elicit immune responses, and the limited understanding of bacteriophage pharmacodynamics in mammalian systems adds further complexity to their therapeutic use [40].

A compelling example is the crAss001 bacteriophage, classified as lytic but capable of long-term coexistence with its Bacteroides host in liquid culture without eliminating the bacterial population [41]. This observation aligns with the concept of pseudolysogeny or carrier state, where a stable equilibrium is maintained despite the absence of lysogeny genes. The persistence of crAss-like bacteriophages in the gut, even after fecal microbiota transplantation, supports this view. It has been proposed that such stability might involve cryptic mechanisms, possibly through host-encoded transposons or quorum-sensing pathways, allowing bacteriophages to persist without classical integration machinery [41,42].

Further supporting this idea is φPDS1, a lytic bacteriophage infecting *Parabacteroides distasonis*, which demonstrates efficient replication and plaque formation but fails to clear its host in liquid cultures. Over time, a significant rise in resistant bacterial subpopulations (~22% to ~95%) was observed, likely due to phase-variable expression of surface receptors. This results in a heterogeneous bacterial population, with some cells being permissive and others resistant, enabling prolonged bacteriophage-host coexistence [39]. Such findings emphasize that bacteriophage persistence may not always require lysogeny but can instead result from selective pressures and population-level adaptations.

Interestingly, despite extensive research, no known bacteriophage has successfully eradicated its host population in natural settings. This suggests that stable coexistence, rather than eradication, might be a more evolutionarily favored strategy [42]. Some bacteriophages, like VP882, can even sense host population density via quorum-sensing systems and modulate their lytic behavior accordingly, indicating a higher level of environmental responsiveness than previously assumed [42].

Finally, interactions between bacteriophages, bacteria, and the mammalian host extend far beyond simple lysis. In murine models, immune-mediated inflammation has been shown to induce probacteriophage activation, exacerbating disease through enhanced lysogenic conversion. In contrast, host vaccination reduced this bacteriophage-induced pathogenicity without impacting bacterial load [43]. These findings highlight that bacteriophages can shape host immunity and disease outcomes, whether lytic or temperate, reinforcing the need to account for bacteriophage–host–immune system dynamics in therapeutic and ecological contexts [44]. Together, these observations underscore the complexity of bacteriophage biology *in vivo*. Even bacteriophages traditionally classified as lytic may exhibit complex population-level behaviors, including cryptic persistence and dynamic balancing with bacterial hosts, which may influence their application in clinical settings and challenge the conventional dichotomy between lytic and temperate lifestyles [45].

The toxin-like gene in Halo KS-7 is proposed to serve a beneficial role in enhancing the bacteriophage’s ability to infect and lyse bacterial hosts. This toxin may function analogously to bacteriocins, antimicrobial proteins that disrupt bacterial membranes or interfere with vital intracellular processes. The identified colicin-like motif suggests that this gene could act similarly to bacteriocins, a class of proteins known for pore-forming and bactericidal activities [46].

Many bacteriophage-derived elements, such as phage tail-like bacteriocins (tailocins), are structurally and functionally analogous to bacteriocins and confer antagonistic activity against competing bacteria, supporting the notion that certain phage-encoded genes may serve as auxiliary antibacterial tools [47]. This activity likely complements the bacteriophage’s primary lytic machinery, including holins that permeabilize the bacterial membrane and facilitate endolysin-mediated cell wall degradation, thereby enhancing the bacteriophage’s capacity to rapidly kill target bacteria through coordinated membrane disruption and cell wall attack [48].

Moreover, such toxin-like auxiliary genes may reflect evolutionary strategies that favor more precise and efficient bacterial killing mechanisms, potentially contributing to phage fitness and ecological success [49]. In this context, Halo KS-7 may represent a bacteriophage therapy tool optimized for selective bacterial eradication while minimizing disruption to the host microbiome.

Phage-encoded virulence factors, including toxin-like genes, have been proposed to enhance phage Darwinian fitness by acting as ecosystem-modifying agents that influence bacterial competition and phage spread in heterogeneous microbial environments [50]. Overall, the toxin-like gene in Halo KS-7 appears to offer potential advantages for enhancing infection efficiency and bacterial lysis, although potential limitations or undesirable effects may arise, particularly if the toxin’s action is not fully controlled in therapeutic applications. Given Halo KS-7’s robust genetic and functional capabilities, any limitations associated with this gene could potentially be addressed using precise gene-editing techniques. Approaches such as CRISPR-Cas or recombineering could remove or modify the toxin gene while preserving the bacteriophage’s lytic activity and antibacterial efficacy, enabling safe and effective clinical application against multidrug-resistant bacterial infections [51]. Our findings accord with and extend recent research on *Klebsiella* bacteriophages. For instance, Peng et al. (2025) [52] isolated a lytic bacteriophage vB_Kp_XP4 that targets hypervirulent K1 *K. pneumoniae*, noting its potent activity and absence of undesirable genes – similar to Halo KS-7 in its strictly lytic nature. Mirza et al. (2025) [53] characterized bacteriophage vbKpUKJ_2 from hospital sewage, which showed a broad host range (infecting ~43% of tested *K. pneumoniae* isolates) and high thermal/pH stability, aligning with Halo KS-7’s robustness. Unlike vbKpUKJ_2 (a Drexlerviridae siphovirus with ~45 kb genome) [53], Halo KS-7 is a myovirus with a slightly larger genome (58.716 kb) and likely a different evolutionary origin. The Halo KS-7 phage demonstrated attributes relevant to clinical therapy and food safety applications. Similar findings were reported by Chen et al. (2023) [54], who characterized bacteriophage vB_KpP_HS106 against *K. pneumoniae* K2, showing stability across pH 4–12 and temperatures from 4–50°C, comparable to Halo KS-7’s stability profile. While vB_KpP_HS106 was primarily studied for food safety purposes, Halo KS-7’s characteristics suggest the potential for broader applications, including clinical therapy and food safety. A distinctive aspect of Halo KS-7 is its halophilic adaptation. Bacteriophages requiring or tolerating high salt are more commonly associated with halophilic bacterial hosts (e.g., *Halomonas*, *Vibrio* in marine settings) than *Klebsiella*. Halo KS-7’s survival in up to 10–15% NaCl without losing much activity is remarkable; by comparison, most enteric bacteriophages are significantly inactivated beyond ~5% NaCl due to osmotic damage to the capsid. This property might translate into advantages for therapeutic formulation. For example, bacteriophage preparations often contain stabilizers like salts or sugars. Halo KS-7 might remain potent in the broader range of formulations or specific infection sites (such as hypertonic cystic fibrosis sputum). While halotolerance is not a typical requirement for bacteriophage therapy, bacteriophages generally demonstrate a broad range of physicochemical stress tolerances, including survival at high salt concentrations, varied pH ranges, and elevated temperatures, underscoring their capacity to adapt to challenging environments and implying possible correlations with other desirable traits such as desiccation resistance or shelf stability [55].

The bacteriophage genome encodes several auxiliary enzymes that enhance infectivity and offer promising therapeutic applications. Among them, the holin gene is critical in orchestrating bacterial lysis. Holins form-controlled pores in the bacterial inner membrane, allowing them to reach the peptidoglycan layer and initiate cell wall degradation. This precise timing and mechanism significantly improve lysis efficiency and accelerate the bacteriophage replication cycle, which is advantageous in therapeutic settings [56].

The Rz-like process further supports the lytic spanin, facilitating the final step of bacterial cell lysis by disrupting the outer membrane. Complete membrane disruption ensures full bacterial collapse, minimizing the survival of residual or partially lysed cells, which is a key factor in effective infection clearance [57].

Additionally, the bacteriophage encodes a MazG-like pyrophosphatase, an enzyme known to interfere with bacterial stress responses. By hydrolyzing nucleotide alarmones such as (p)ppGpp, this enzyme disrupts the stringent response, which is a defense mechanism activated under nutrient limitation or antibiotic exposure. This interference may sensitize bacteria to bacteriophage attack and reduce the formation of persister cells, thereby enhancing the overall efficacy of bacteriophage therapy [15].

The presence of a bacteriophage-encoded RNA polymerase further underscores its adaptability. This enzyme allows the transcription of bacteriophage genes independently of the host’s transcriptional machinery, which is advantageous when the host is under metabolic stress or suppresses foreign gene expression [58].

Lastly, the ribonucleotide reductase gene converts ribonucleotides into deoxyribonucleotides, ensuring a sufficient supply of DNA precursors for viral replication. This is especially beneficial in nutrient-limited environments such as biofilms or infected tissues, where nucleotide availability may be restricted [59].

Together, these enzymes constitute a strategic genetic arsenal that enhances bacteriophage replication, promotes efficient host cell lysis, and improves resilience under stress. Their presence suggests strong potential for therapeutic use, especially in targeting persistent or MDR bacterial infections.

Intriguingly, the Halo KS-7 genome harbors three tRNA (tRNA^Tyr^, tRNA^Pro^, and tRNA^Asn^) genes, suggesting an adaptive strategy to optimize translational efficiency when host tRNA pools are limiting. The bacteriophage can sustain high‐level protein synthesis during infection by supplying its tRNAs, promoting rapid replication and potent lytic activity. Although minimal-genome approaches often eschew accessory elements, recent work shows that standalone tRNA genes pose negligible biosafety risks when unlinked to lysogeny or mobile elements. Because Halo KS-7 lacks integrase and follows a strictly lytic lifecycle, these tRNAs likely represent evolutionary fine-tuning for efficient host takeover rather than a hazard. Such features underscore the therapeutic potential of Halo KS-7 against CRKP, especially in challenging clinical contexts.

The application of an AI-assisted annotation pipeline facilitated rapid functional hypothesis generation for several hypothetical proteins encoded by Halo KS-7. For example, one small ORF lacking clear homology in conventional BLAST searches was flagged by the pipeline as a potential DNA-binding protein based on sequence features consistent with a helix–turn–helix motif. While such motifs can also be identified using established profile-based tools (e.g., HHpred), the value of the AI-based approach in this context lies in its ability to automate large-scale screening and prioritize candidate functions without manual interrogation of individual ORFs. Importantly, AI-assisted predictions were treated as exploratory and complementary, requiring confirmation through conventional bioinformatic or experimental validation [14].

Halo KS-7 meets several key criteria for a therapeutic bacteriophage candidate. First, it is strictly lytic and lacks genes for lysogeny, minimizing safety concerns [52,53]. Second, it has a relatively broad action against diverse *K. pneumoniae* strains, including MDR and hypervirulent types, which is essential given the genetic diversity of *K. pneumoniae* clinical isolates [51]. Third, the bacteriophage genome encodes several enzymes, such as holins, spanins, MazG-like pyrophosphatase, RNA polymerase, and ribonucleotide reductase that collectively enhance its ability to infect and lyse bacterial cells. These enzymes enable efficient membrane disruption, suppress bacterial stress responses, and replication of bacteriophage under nutrient-limited or stressful conditions [15,59,60–62]. Their presence suggests the bacteriophage is well-suited for therapeutic applications, potentially maintaining stability and activity across various infection environments and delivery routes such as inhalation or topical use.

Fourth, its stability in a range of conditions implies it could be formulated for various routes of administration (inhalation for pneumonia, topical for wound infections, etc.) without rapid inactivation.

Recent *in vivo* studies give optimism that bacteriophages like Halo KS-7 could be effective. For example, Gan et al. (2022) [63] demonstrated that two lytic bacteriophages could rescue mice from fatal *K. pneumoniae* pneumonia, significantly reducing bacterial load and inflammation. Similarly, Duarte et al. (2022) [64] reported successful compassionate use of bacteriophages in a human patient with recurrent *Klebsiella* urinary infection. These cases, alongside rigorous laboratory evaluations, suggest that bacteriophages are moving from bench to bedside for *K. pneumoniae*. Halo KS-7’s broad host range would be advantageous for treating infections where the specific capsular type is unknown or mixed *Klebsiella* populations are present. In a practical scenario, Halo KS-7 could be used as part of a bacteriophage cocktail to preempt resistance – combining it with other bacteriophages targeting complementary receptors. Indeed, bacteriophage cocktails have broadened the overall killing spectrum and delayed the emergence of bacteriophage-resistant mutants [65].

While this study provides a comprehensive analysis of Halo KS-7, there are some limitations. We did not perform *in vivo* experiments, so the bacteriophage’s efficacy and immunogenicity in animal infection models remain to be tested. Given its promising *in vitro* profile, testing Halo KS-7 in a mouse pneumonia model, similar to Gan et al. and Li et al., [63,66], would be a logical step to confirm therapeutic potential and safety (e.g., absence of immunopathology).

From a genomic standpoint, Halo KS-7 underscores how much bacteriophage genetic diversity remains undiscovered. The AI pipeline flagged several unique ORFs; characterizing these (via knockout mutagenesis or structural studies) could reveal novel functions. One speculative but intriguing ORF encodes a small protein of 90 amino acids with a repeated motif; such proteins sometimes act as inhibitors of host processes (e.g., protease inhibitors or nucleotide sequestration proteins). Uncovering any anti-host mechanisms could augment our understanding of how bacteriophages subvert *Klebsiella* during infection. Furthermore, exploring the relationship of Halo KS-7 with the rare bacteriophages it is related to (like the “vB_KpnM_Saline”) could help define a new bacteriophage genus. Given the mosaic nature of bacteriophage genomes, the presence or absence of specific functional modules such as lysis-related or host takeover genes shows the importance of analyzing bacteriophage genes individually and in the context of the complete genome.

## 5. Conclusion

Comprehensive characterization of Halo KS-7 reveals a potent lytic bacteriophage with distinctive halophilic adaptation and a broad host range. Isolated from a clinical setting and thoroughly examined through laboratory assays and an AI-assisted genomic annotation pipeline, Halo KS-7 represents a novel addition to the growing repertoire of bacteriophages with translational promise. Genomic analysis confirmed the absence of antibiotic-resistance, integrase, or recombinase gene, underscoring its strictly lytic lifecycle and lack of lysogenic potential. Intriguingly, the genome also harbors three tRNA genes (tRNA^Tyr^, tRNA^Pro^, and tRNA^Asn^), which likely optimize translational efficiency when host tRNA pools are limited, sustaining rapid protein synthesis and enhancing lytic activity. The presence of a toxin gene and auxiliary factors such as MazG-, like pyrophosphatase and HNH endonucleases, further suggests built-in enhancements to bacterial killing without promoting HGT or antimicrobial resistance (AMR), reinforcing its therapeutic safety. Finally, the application of AI in genomic annotation was instrumental in rapidly distinguishing both beneficial and undesirable elements, showcasing the power of computational tools in modern bacteriophage research.

As AMR continues to escalate, bacteriophages like Halo KS-7 offer a timely and targeted alternative for infection control. Future directions will focus on assessing its efficacy *in vivo* infection models, exploring formulation strategies, and evaluating its role within bacteriophage cocktails or as a source of recombinant enzymes. Incorporating such bacteriophages into therapeutic pipelines and clinical trials will be crucial to realizing their clinical impact. Our findings contribute to the broader understanding of bacteriophage diversity and function, laying the groundwork for further development of Halo KS-7 in combating critical *K. pneumoniae* infections.
